# *PRKAA2* variation and the clinical characteristics of patients newly diagnosed with type 2 diabetes mellitus in Yogyakarta, Indonesia

**DOI:** 10.2478/abm-2021-0021

**Published:** 2021-08-20

**Authors:** Dita Maria Virginia, Mae Sri Hartati Wahyuningsih, Dwi Aris Agung Nugrahaningsih

**Affiliations:** Department of Pharmacology and Therapy, Faculty of Medicine, Public Health and Nursing, Universitas Gadjah Mada, Yogyakarta 55281, Indonesia; Faculty of Pharmacy, Universitas Sanata Dharma, Yogyakarta 552181, Indonesia; Center of Genetic Study, Faculty of Medicine, Public Health and Nursing, Universitas Gadjah Mada, Yogyakarta 55281, Indonesia

**Keywords:** genetic variation, Indonesia, *PRKAA2*, type 2 diabetes mellitus

## Abstract

**Background:**

Adenosine monophosphate (AMP)-activated protein kinase (AMPK; EC 2.7.11.31) enzymes play a pivotal role in cell metabolism. They are involved in type 2 diabetes mellitus (T2DM) pathogenesis. Genetic variation of *PRKAA2* coding for the AMPK α2 catalytic subunit (AMPKα2) is reported to be associated with susceptibility for T2DM.

**Objectives:**

To determine the association between *PRKAA2* genetic variations (rs2796498, rs9803799, and rs2746342) with clinical characteristics in patients newly diagnosed with T2DM.

**Methods:**

We performed a cross-sectional study including 166 T2DM patients from 10 primary health care centers in Yogyakarta, Indonesia. We measured fasting plasma glucose, hemoglobin A1c, serum creatinine, glomerular filtration rate, blood pressure, and body mass index as clinical characteristics. *PRKAA2* genetic variations were determined by TaqMan SNP genotyping assay. Hardy–Weinberg equilibrium was calculated using χ^2^ tests.

**Results:**

There was no difference in clinical characteristics for genotypes rs2796498, rs9803799, or rs2746342 (*P* > 0.05). No significant association was found between *PRKAA2* genetic variations and any clinical feature observed. Further subgroup analysis adjusting for age, sex, and waist circumference did not detect any significant association of *PRKAA2* genetic variations with clinical characteristics (*P* > 0.05).

**Conclusion:**

*PRKAA2* genetic variation is not associated with the clinical characteristics of Indonesian patients with newly diagnosed T2DM.

Type 2 diabetes mellitus (T2DM) is a degenerative disease affecting morbidity and mortality. The prevalence of T2DM is increasing worldwide, and >60% of patients with T2DM are located in Asia. It is predicted that by 2035, the incidence of T2DM in Indonesia will be 2 times higher than it was in 2013. Indonesia has the second-highest ranking of T2DM prevalence among Western Pacific Region countries [1, 2]. Our previous study found that T2DM prevalence was higher in the Sleman population of Yogyakarta, a densely populated province on the island of Java in Indonesia [3].

It is widely known that T2DM is a disease with multi-factorial etiology, including environmental and multigenic factors that are involved in T2DM pathogenesis [4]. The racial, ethnic, social, economic, and cultural differences of Pacific Islanders, including in Indonesia, have created complex gene-environment interactions [1]. It is noteworthy that a study conducted in the United States population found that T2DM genetic risk also increased the risk of mortality [5].

Previous studies have investigated many genes that correlate with T2DM risk. Genome-wide association studies (GWASs) are some of the largest to explore the association of genetics with disease risk, including for T2DM. A GWAS meta-analysis found approximately 143 variants and risk alleles that could increase risk of T2DM [6]. While early GWASs were focused on Europe, a cohort study in Singapore has observed multiethnic populations in Southeast Asia, engaging Malay, Chinese, and Asian Indian patients. Despite the study's success in discovering variants that have an association with T2DM risk, it was limited in that a study of disease risk based exclusively on specific populations was still required [7]. Heritability of T2DM is reported to be about 20%–80% from progeny or twin studies [8, 9], but T2DM genetic risk is not always inherited, and is well-known as “missing heritability”. Gene–environment and gene–gene interaction might contribute to missing heritability of T2DM [10, 11]. Accordingly, detection in a specific population is better to reduce missing heritability risk, which in this present study has focused only on Indonesian patients newly diagnosed with T2DM in Yogyakarta.

Variation of the genes that contribute to glucose and fat metabolism may contribute to the increasing of T2DM risk [12]. *PRKAA2* (NCBI gene ID: 5563), which encodes protein kinase adenosine monophosphate (AMP)-activated (AMPK; EC 2.7.11.31) α2 catalytic subunit (AMPKα2) is a gene that regulates glucose and lipid cellular metabolism. Accordingly, it is a promising gene candidate to detect T2DM risk. AMPK is induced when the cellular energy levels are below normal and has a pivotal role in regulating energy metabolism in adipose tissue, skeletal muscle, and the liver. AMPK signaling will stimulate glucose uptake in skeletal muscle, lipid oxidation in adipose tissues, and attenuate glucose production in the liver [13,14,15]. A review found a close association between dysregulation of AMPK and insulin resistance [16]. More recent review suggests AMPK is involved in blood pressure and renal function [17]. Several articles have proposed that AMPK is a promising therapeutic target for T2DM [18,19,20].

An association between genetic variations of *PRKAA2* and risk of T2DM has been observed among Japanese [21, 22] and Han Chinese [23,24,25] populations. Those studies explored 18 single nucleotide polymorphisms (SNPs), and found that only 4 SNPs are correlated with T2DM risk. Additionally, a review stated that *PRKAA2* genetic variation has a relationship with diabetic kidney disease [26]. Our present study explored 3 candidate SNPs that have been proven to be associated with T2DM: rs2796498, rs9803799, and rs2746342. Li et al. found that rs2796498 has a significant association with susceptibility of T2DM [25]. SNP rs9803799 is one of the *PRKAA2* genetic variations that has an impact on metformin pharmacodynamics relating to reducing T2DM progression [27]. A number of studies of SNP rs2746342 found its significant association with T2DM or T2DM nephropathy risk [24, 25].

Fasting plasma glucose (FPG) and glycated hemoglobin A1c (HbA1c) are the most widely used biomarkers to diagnose T2DM based on the American Diabetes Association (ADA) criteria. Our study implies that the diagnostic tool could be enhanced by merging these with analyses of genetic variation. Meanwhile, T2DM is a degenerative disease that could lead to complications, so it is essential to examine clinical characteristics as conventional risk factors. Body mass index (BMI), waist circumference, elevated blood pressure, and hyperglycemia could augment T2DM severity and increase the risk of T2DM complications [28]. Additionally, reduced renal function is a common T2DM complication marked by declining levels of the estimated glomerular filtration rate (eGFR) [29, 30]. Accordingly, those factors were observed in our study. The association of *PRKAA2* genetic variations with clinical characteristics among Indonesian patients with T2DM, especially in Yogyakarta, has not yet been determined. Therefore, the present study aimed to investigate the association of *PRKAA2* genetic variations, in particular, rs2796498, rs9803799, and rs2746342, with clinical features among newly diagnosed T2DM patients in Indonesia. To our knowledge, this study is the first to report any associations with an Indonesian population, specifically in patients newly diagnosed with T2DM who live in Yogyakarta Province.

## Methods

### Study design, setting, and participants

In the present cross-sectional study, we recruited 190 patients with suspected T2DM from 10 primary health care (PHC) centers located in Yogyakarta, Indonesia, between June 2019 and July 2020. The study size was calculated using a 5% level of significance and power of 80%, while the expected prevalence of T2DM in rs2746342 of TG genotype was 49% and in rs2746342 of GG genotype was 26% [24], and we applied 2 equal groups. Therefore, using the Fleiss formula, we ascertained that a sample of 156 patients was required.

The inclusion criteria as in the previous study were patients with age 20–75 years, Indonesian, and a diagnosis by a physician of T2DM based on the ADA criteria, which are FPG ≥126 mg/dL or HbA1c ≥6.5%. We conducted the laboratory tests to determine concentrations of FPG, HbA1c, and creatinine serum for all participants. Any participant who did not have laboratory test results was excluded. A nurse obtained blood pressure by direct measurement. A nutritionist in the PHCs conducted anthropometric measurements, including height, weight, and waist circumference. We calculated BMI by dividing weight (kg) by height (m^2^) and obtained age and sex data from the patients’ medical records.

The study protocol was approved by the Medical and Health Research Ethics Committee (MHREC), Faculty of Medicine, Public Health and Nursing, Universitas Gadjah Mada – Dr. Sardjito General Hospital in Yogyakarta, Indonesia (reference No. KE/FK/0633/EC/2019) as recognized by the FERCAP and complied with the ethical principles of the contemporary revision of the Declaration of Helsinki and other international and national guidelines on ethical standards and procedures for research on human beings. All participants signed an informed consent form to participate in this study. This study is reported according to STREGA reporting guidelines, extended from the STROBE statement [31].

### Variables

Based on previous studies, we selected 3 SNPs that have minor allele frequency (MAF) > 10%: rs2796498, rs9803799, and rs2746342. These SNPs have been identified among Han Chinese and a U.S. population of various ancestries [24, 27, 32]. Dependent variables were clinical characteristics of patients newly diagnosed with T2DM including age, BMI, waist circumference, blood pressure, FPG, HbA1c, and renal function. Lifestyle, age, and sex might influence the results besides the effect of genetic variation as a potential bias. Therefore, for the present study we conducted further analysis adjusting for sex, age, and waist circumference.

### Data sources measurement

#### Clinical measurements

After an overnight fast, an analyst at the PHC collected a venous blood sample into a tube containing ethylenediaminetetraacetic acid (EDTA). Blood sample parameters were measured on the same day as the sample was collected. All laboratory tests were measured by Prodia Laboratory Instruments (Yogyakarta, Indonesia). FPG was measured using a hexokinase method, and serum creatinine was measured using an enzymatic method. HbA1c was quantified by ion-exchange high-performance liquid chromatography D-10. eGFR was calculated using a Chronic Kidney Disease Epidemiology Collaboration (CKD-EPI) formula for non-Black populations and included serum creatinine (mg/dL).

#### PRKAA2 genetic variation analysis

Blood samples of participants were collected by venipuncture in 1.5 mL tubes containing EDTA and stored at −20 °C in a freezer. A genomic DNA sample was isolated from the whole blood–EDTA sample using a Genomic DNA Mini Kit (Blood) (RA501500; Genaid, Taiwan) according to the manufacturer's instructions and stored at −80 °C. The genetic variations were genotyped using TaqMan SNP genotyping assays and Applied Biosystems qPCR 7500 Fast Real-Time PCR System located at the Faculty of Medicine, Public Health and Nursing, Universitas Gadjah Mada. The total reaction volume was 10 μL. Details of all TaqMan primers and probes (catalog Nos. 4351379 and 4403311), and conditions for genotyping, are available upon request. Context sequences (VIC/FAM) for TaqMan assay are listed in **Table 1** [33]. All reactions were performed with the following cycle parameters: 40 cycles at hold 95 °C for 20 s, at denaturing 95 °C for 3 s, and followed by annealing 60 °C for 30 s. We assigned the genotyping data in batches.

**Table 1:** Context sequence (VIC/FAM) rs2796498, rs9803799, and rs2746342

SNP ID^*^	**Context sequence (VIC/FAM dye)**
rs2796498	CTGTAACAGTGTTAGTGATTTAAAC**[A/G]** GAGAGAGCAACCTTACCCTTTCAGT
rs9803799	TAAATACAGGGTTTATATCCCCACA**[G/T]** TCAATGTAAATTCCTTTTTTTAAAA
rs2746342	AGAGAGGCTAAGATGCAGGCTGTAC**[G/T]** CTGGGTAGCCATGTACTCAGTTGTA

* TaqMan SNP Genotyping Assays by Applied Biosystems (Thermo Fisher Scientific).

SNP, single point mutation.

### Statistical analysis

Descriptive analysis was conducted to analyze the baseline characteristics of the participants. Clinical characteristics of participants with different genotypes in each SNP were compared. First, we performed a test of homogeneity to determine whether to use a one-way ANOVA or Kruskal–Wallis test. The mean difference of eGFR in rs2796498 and serum creatinine in rs2796498 and rs9803799 was *P* < 0.05 in the test of homogeneity, so they were subsequently analyzed using Kruskal–Wallis tests. Hardy–Weinberg equilibrium was calculated using χ^2^ tests. Association between *PRKKA2* genetic variations and clinical characteristics using bivariate logistic regression analysis requires alteration from numeric to categorical data. Therefore, FPG, HbA1c, and serum creatinine were grouped by mean in baseline characteristics. Blood pressure ≥140/≥90 mmHg was categorized as high blood pressure. Participants who had BMI >25 kg/m^2^ were classified as obese. According to the CKD definition, declining renal function was defined as eGFR <60 mL/min/1.73 m^2^. We used 3 consecutive models: the first was a nonadjusted model, the second model was adjusted by age and sex, and the third model was adjusted by age, sex, and waist circumference. Two-tailed statistical tests were used. The association was presented as an odds ratio (OR) with 95% confidence interval (CI) and the level of statistical significance was set at *P* < 0.05. Data were analyzed using IBM SPSS Statistics for Windows software (version 25).

## Results

We included 166 patients newly diagnosed with T2DM in the present study. We had excluded 20 participants who had FPG <126 mg/dL or HbA1c <6.5% from the initial 190 patients. We had also excluded 4 participants because of lysis of their blood sample. Genotypes of all participants were analyzed successfully. The baseline characteristics of the participants are presented in **Table 2**. The mean age of the patients in our sample was 54.0 ± 9.7 years, and 70.5% were female. Mean blood pressure in our participants was categorized as prehypertension with systolic ≥120 mmHg and diastolic ≥80 mmHg. The mean BMI and waist circumference indicated that our population tended to be overweight. In our present study the patients newly diagnosed with T2DM tended to have elevated blood pressure, but normal renal function as determined by eGFR. The mean of HbA1c was high for patients newly diagnosed with T2DM.

**Table 2:** Baseline characteristics of the patients with T2DM

**Characteristics**	**(n = 166)**
Age (years)	54.0 ± 9.7
Sex (female)	117 (70.5)
Systolic blood pressure (mmHg)	130.4 ± 18.7
Diastolic blood pressure (mmHg)	81.1 ± 8.7
BMI (kg/m^2^)	25.0 ± 4.0
Waist circumference (cm)	87.6 ± 9.2
FPG (mg/dL)	189.0 ± 71.2
HbA1c (%)	9.61 ± 2.32
CrSr (mg/dL)	0.89 ± 0.80
eGFR (mL/min/1.73 m^2^)	91.6 ± 26.7

Continuous variables are presented as mean ± standard deviation, sex is presented as n (%).

BMI, body mass index; CrSr, serum creatinine; eGFR, estimated glomerular filtration rate; FPG, fasting plasma glucose; HbA1c, hemoglobin A1c; T2DM, type 2 diabetes mellitus.

**Figure 1:**
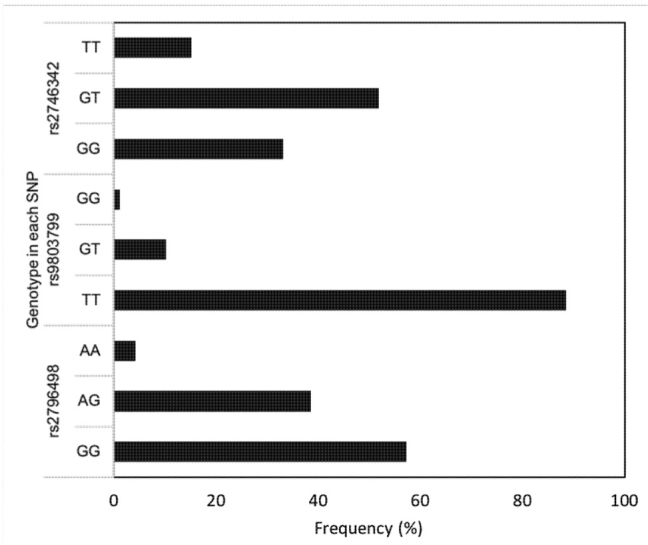
Distribution of *PRKAA2* genotype rs2796498, rs9803799, and rs2746342 in patients newly diagnosed with T2DM in Yogyakarta, Indonesia; *PRKAA2* (NCBI gene ID: 5563), which encodes protein kinase adenosine monophosphate (AMP)-activated (EC 2.7.11.31) α2 catalytic subunit (AMPKα2); SNP, single nucleotide polymorphism; T2DM, type 2 diabetes mellitus.

Genotype frequencies of the *PRKAA2* genetic variations are described in **Figure 1**. The genotype frequencies of *PRKAA2* rs2796498, rs9803799, and rs2746342 genetic variations were in Hardy–Weinberg equilibrium (*P* = 0.35; *P* = 0.08; and *P* = 0.36, respectively). Only 4.2% wild type of rs2796498 and 1.2% wild type of rs980799 were found in the genotyping results of this study.

The mean differences of clinical characteristics and genotype frequencies are listed in **Table 3**. Patients with a mutant genotype (TT) of rs2746342 tended to have a higher BMI than those with the wild type. Waist circumference was greater in patients with GT of rs9803799 than in those with TT or GG. Systolic blood pressure in rs2796498 was higher in patients with the wild type (AA), while in those with rs9803799 it was higher than in those with the mutant (TT) type. HbA1c was lower in patients with the wild-type genotype both for rs2796498 and rs9803799. Serum creatinine was higher in patients with the mutant genotype of rs2796498 and rs9803799, but it was higher in heterozygotes for rs2746342. The means of patients’ age, sex, blood pressure, BMI, waist circumference, and FPG, HbA1c, and serum creatinine concentrations, and eGFR were not significantly different between groups with rs2796498, rs9803799, and rs2746342.

**Table 3:** Clinical characteristics of patients with T2DM patients based on *PRKAA2*genetic variation

**Clinical Characteristic**	**rs2796498 (HWE 0.35)** **n (%)**			***P***	**rs9803799 (HWE 0.08)** **n (%)**			***P***	**rs2746342 (HWE 0.36)** **n (%)**			***P***
		
	**GG 95 (57.2)**	**AG 64 (38.6)**	**AA 7 (4.2)**		**TT 147 (88.6)**	**GT 17 (10.2)**	**GG 2 (1.2)**		**GG 55 (33.1)**	**GT 86 (51.8)**	**TT 25 (15.1)**	
Age (years)	53.3 ± 9.5	54.7 ± 10.2	55.7 ± 10.1	0.60	54.1 ± 9.6	53.7 ± 11.3	45.0 ± 2.8	0.42	54.3 ± 9.6	53.7 ± 9.8	54.0 ± 10.2	0.94
BMI (kg/m^2^)	24.95 ± 3.78	24.83 ± 4.20	27.14 ± 5.40	0.35	25.06 ± 4.03	24.53 ± 4.09	24.50 ± 3.54	0.87	24.60 ± 4.20	25.23 ± 3.69	25.09 ± 4.73	0.66
WC (cm)	87.4 ± 8.7	87.6 ± 10.1	91.0 ± 8.1	0.61	87.4 ± 9.5	90.1 ± 6.5	85.5 ± 3.5	0.49	86.6 ± 9.2	88.2 ± 9.6	88.0 ± 7.9	0.58
SBP (mmHg)	129.5 ± 19.1	131.3 ± 18.5	136.4 ± 15.0	0.57	131.1 ± 18.9	125.7 ± 14.8	124.0 ± 33.9	0.47	127.4 ± 19.7	131.8 ± 18.2	132.9 ± 17.7	0.31
DBP (mmHg)	81.2 ± 8.9	81.1 ± 8.5	80.4 ± 9.6	0.98	81.3 ± 8.2	79.6 ± 12.3	82.0 ± 17.0	0.74	80.0 ± 9.5	81.8 ± 8.6	81.3 ± 7.2	0.51
FPG (mg/dL)	188.8 ± 72.2	191.8 ± 70.8	167.9 ± 68.1	0.70	188.5 ± 70.0	195.5 ± 83.1	196.0 ± 103.2	0.97	186.5 ± 75.2	189.6 ± 68.8	192.7 ± 73.1	0.93
HbA1c (%)	9.65 ± 2.30	9.61 ± 2.35	8.97 ± 2.43	0.76	9.61 ± 2.25	9.66 ± 2.85	8.9 ± 3.40	0.91	9.42 ± 2.30	9.79 ± 2.32	9.39 ± 2.89	0.58
CrSr (mg/dL)	0.81 ± 0.49	1.04 ± 1.13	0.66 ± 0.10	0.48	0.87 ± 0.62	1.13 ± 1.75	0.67 ± 0.13	0.37	0.83 ± 0.52	0.97 ± 1.01	0.80 ± 0.33	0.48
eGFR (mL/min)	94.0 ± 24.7	87.5 ± 30.2	96.6 ± 13.1	0.66	91.5 ± 26.2	90.7 ± 32.6	111.5 ± 3.5	0.57	92.1 ± 24.6	90.9 ± 29.0	93.0 ± 23.7	0.93

Data were analyzed using a one-way ANOVA or Kruskal–Wallis test, as appropriate.

BMI, body mass index; CrSr, serum creatinine; DBP, diastolic blood pressure; eGFR, estimated glomerular filtration rate; FPG, fasting plasma glucose; HbA1c, hemoglobin A1c; HWE, Hardy–Weinburg equilibrium; SBP, systolic blood pressure; T2DM, type 2 diabetes mellitus; WC, waist circumference.

We also analyzed the association between *PRKKA2* genetic variations and clinical characteristics. As shown in **Table 4**, when the GG, TT, and GG in rs2796498, rs9803799, and rs2746342, respectively, were used as a reference, there was no significant association between *PRKKA2* genetic variation and clinical characteristics (*P* > 0.05). We did not find any significant association in dominant, recessive, or allele models (*P* > 0.05). However, we found that AG, dominant model, and A allele in rs2796498 tended to increase the risk of higher FPG, higher CrSr, and eGFR <60 mL/min/m^2^ (*P* > 0.05). By contrast, GT, dominant model, and G allele in rs9803799 reduced the risk of higher HbA1c and higher blood pressure, but increased the risk of eGFR <60 mL/min/m^2^. Meanwhile, only the recessive model in rs2746342 indicated any reduction of the risk for higher FPG, higher HbA1c, higher CrSr concentration, and eGFR <60 mL/min/m^2^, but was not significantly associated (*P* > 0.05).

**Table 4:** Association between *PRKAA2* genetic variation and clinical characteristics of patients with T2DM

**Genotype**	**OR (95%CI)**					

	**FPG**	**HbA1c**	**CrSr**	**eGFR**	**Blood pressure**	**Obesity status**
rs2796498						
GG	1 (Reference)					
AG	1.24 (0.66–2.34)	0.90 (0.48–1.71)	1.74 (0.86–3.51)	2.51 (0.96–6.54)	0.98 (0.51–1.92)	1.18 (0.63–2.23)
AA	0.99 (0.21–4.69)	0.46 (0.09–2.51)	<0.01 (<0.01–NA)	<0.01 (<0.01–NA)	1.41 (0.30–6.68)	1.48 (0.31–6.98)
Dominant (GG vs. AG+AA)	1.21 (0.65–1.26)	0.85 (0.46–1.58)	1.49 (0.75–2.98)	2.21 (0.85–5.74)	1.02 (0.54–1.95)	1.21 (0.65–2.24)
Recessive (GG+AG vs. AA)	0.91 (0.20–4.18)	0.48 (0.09–2.57)	<0.01 (<0.01–NA)	<0.01 (<0.00–NA)	1.42 (0.31–6.56)	1.39 (0.30–6.39)
G allele	1 (Reference)					
A allele	1.13 (0.68–1.87)	0.83 (0.50–1.38)	1.19 (0.64–1.97)	1.47 (0.71–3.04)	1.06 (0.62–1.80)	1.18 (0.71–1.96)

rs9803799						
TT	1 (Reference)					
GT	1.10 (0.40–2.98)	0.86 (0.31–2.38)	0.82 (0.25–2.67)	1.64 (0.43–6.29)	0.55 (0.17–1.76)	0.90 (0.33–2.46)
GG	1.23 (0.08–19.99)	1.23 (0.08–19.99)	<0.01 (<0.01–NA)	<0.01 (<0.01–NA)	1.77 (0.11–28.94)	1.01 (0.06–16.51)
Dominant (TT vs. GT+GG)	1.11 (0.42–2.88)	0.89 (0.34–2.35)	0.71 (0.22–2.28)	1.43 (0.38–5.44)	0.63 (0.22–1.86)	0.91 (0.35–2.38)
Recessive (TT+GT vs. GG)	1.22 (0.08–19.78)	1.25 (0.08–20.27)	<0.01 (<0.01–NA)	<0.01 (<0.01–NA)	1.88 (0.12–30.57)	1.03 (0.06–16.66)
T allele	1 (Reference)					
G allele	1.11 (0.46–2.69)	0.93 (0.38–2.27)	0.64 (0.21–1.94)	1.23 (0.35–4.39)	0.73 (0.28–1.94)	0.93 (0.38–2.24)

rs2746342						
GG	1 (Reference)					
GT	1.43 (0.72–2.84)	1.55 (0.78–3.08)	0.95 (0.43–2.06)	2.48 (0.77–7.97)	1.44 (0.70–2.99)	1.90 (0.95–3.77)
TT	1.18 (0.45–3.07)	1.23 (0.49–3.32)	1.65 (0.60–4.56)	1.11 (0.19–6.49)	1.63 (0.60–4.37)	1.39 (0.53–3.59)
Dominant (GG vs. GT+TT)	1.37 (0.71–2.64)	1.48 (0.77–2.86)	1.09 (0.52–2.27)	2.15 (0.68–6.76)	1.48 (0.74–2.98)	1.77 (0.92–3.40)
Recessive (GG+GT vs. TT)	0.95 (0.40–2.23)	0.97 (0.41–2.29)	1.70 (0.70–4.20)	0.59 (0.13–6.16)	1.29 (0.54–3.09)	1.94 (0.40–2.19)
G allele	1 (Reference)					
T allele	1.14 (0.73–1.76)	1.19 (0.76–1.84)	1.21 (0.74–1.97)	1.21 (0.62–2.35)	1.28 (0.81–2.02)	1.27 (0.82–1.97)

AMP, adenosine monophosphate; AMPKα2, AMP-activated protein kinase (EC 2.7.11.31) α2 catalytic subunit; CI, confidence interval; CrSr, serum creatinine; eGFR, estimated glomerular filtration rate; FPG, fasting plasma glucose; HbA1c, hemoglobin A1c; NA, not available; OR, odds ratio; *PRKAA2* (NCBI gene ID: 5563), which encodes AMPKα2; T2DM, type 2 diabetes mellitus.

Even after adjusting for age and sex (**Table 5**), our study did not find any association of *PRKAA2* genetic variations in rs2796498, rs9803799, or rs2746342 with clinical characteristics in the Indonesian population, specifically in Yogyakarta, in patients newly diagnosed with T2DM (*P* > 0.05). Remarkably, the highest association was found in rs2746342 with renal function, both serum creatinine concentration and eGFR, but the association was not significant.

**Table 5:** Multiple regression logistic analysis adjusted for age and sex

**Genotype**	**OR (95%CI)**					

	**FPG**	**HbA1c**	**CrSr**	**eGFR**	**Blood pressure**	**Obesity status**
rs2796498						
GG	1 (Reference)					
AG	1.27 (0.67–2.41)	0.96 (0.50–1.85)	1.79 (0.81–3.94)	2.38 (0.87–6.50)	0.95 (0.48–1.86)	1.20 (0.64–2.28)
AA	1.03 (0.22–4.89)	0.44 (0.08–2.48)	<0.01 (<0.01–NA)	<0.01 (<0.01–NA)	1.27 (0.26–6.14)	1.49 (0.31–7.07)
Dominant (GG vs. AG+AA)	1.24 (0.67–2.32)	0.89 (0.47–1.69)	1.51 (0.70–3.29)	2.08 (0.77–5.67)	0.98 (0.51–1.88)	1.23 (0.66–2.28)
Recessive (GG+AG vs. AA)	0.93 (0.20–4.34)	0.45 (0.08–2.48)	<0.01 (<0.01–NA)	<0.01 (<0.01–NA)	1.30 (0.28–6.13)	1.38 (0.30–6.42)
G allele	1 (Reference)					
A allele	1.15 (0.69–1.92)	0.85 (0.50–1.44)	1.11 (0.59–2.09)	1.34 (0.65–3.00)	1.02 (0.59–1.74)	1.19 (0.72–1.98)

rs9803799						
TT	1 (Reference)					
GT	1.08 (0.39–2.98)	0.79 (0.27–2.27)	0.86 (0.22–3.33)	1.67 (0.39–7.14)	0.54 (0.17–1.74)	0.89 (0.32–2.44)
GG	1.06 (0.06–17.61)	1.07 (0.06–17.93)	<0.01 (<0.01–NA)	<0.01 (0.01–NA)	2.52 (0.15–42.41)	0.96 (0.06–15.95)
Dominant (TT vs. GT+GG)	1.08 (0.41–2.83)	0.81 (0.30–2.21)	0.72 (0.19–2.70)	1.53 (0.37–6.43)	0.65 (0.22–1.91)	0.90 (0.34–2.34)
Recessive (TT+GT vs. GG)	1.05 (0.06–17.44)	1.03 (0.06–18.30)	<0.01 (<0.01–NA)	<0.01 (0.01–NA)	2.66 (0.16–44.71)	0.97 (0.06–16.11)
T allele	1 (Reference)					
G allele	1.07 (0.44–2.61)	0.71 (0.84–2.12)	0.63 (0.18–2.22)	1.38 (0.35–5.39)	0.77 (0.29–2.06)	0.91 (0.37–2.21)

rs2746342						
GG	1 (Reference)					
GT	1.42 (0.71–2.83)	1.55 (0.76–3.14)	0.99 (0.42–2.37)	2.81 (0.83–9.51)	1.48 (0.71–3.09)	1.89 (0.95–3.76)
TT	1.17 (0.45–3.06)	1.30 (0.48–3.47)	1.88 (0.59–5.95)	1.04 (0.16–6.65)	1.67 (0.61–4.54)	1.39 (0.54–3.60)
Dominant (GG vs. GT+TT)	1.36 (0.71–2.63)	1.49 (0.75–2.93)	1.16 (0.51–2.63)	2.34 (0.71–7.71)	1.52 (0.75–3.07)	1.76 (0.91–3.40)
Recessive (GG+GT vs. TT)	0.95 (0.40–2.23)	0.99 (0.41–2.40)	1.89 (0.68–5.26)	0.52 (0.10–2.62)	1.31 (0.54–3.16)	0.94 (0.40–2.21)
G allele	1 (Reference)					
T allele	1.13 (0.73–1.76)	1.20 (0.76–1.87)	1.28 (0.73–2.21)	1.22 (0.61–2.45)	1.03 (0.82–2.06)	1.27 (0.82–1.97)

CI, confidence interval; CrSr, serum creatinine; eGFR, estimated glomerular filtration rate; FPG, fasting plasma glucose; HbA1c, hemoglobin A1c; NA, not available; OR, odds ratio.

Our third model is presented in **Table 6**. The present study failed to discover any significant association of *PRKAA2* genetic variation with clinical characteristics. It is notable that the recessive model of rs27476342 had a lower OR in association with FPG, HbA1c, eGFR, and obesity status compared with the genotype or dominant models.

**Table 6:** Multiple regression logistic analysis adjusted for age, sex, and waist circumference

**Genotype**	**OR (95%CI)**					

	**FPG**	**HbA1c**	**CrSr**	**eGFR**	**Blood pressure**	**Obesity status**
rs2796498						
GG	1 (Reference)					
AG	1.29 (0.67–2.45)	0.97 (0.50–1.87)	1.79 (0.81–3.96)	2.38 (0.87–6.49)	0.92 (0.46–1.84)	1.31 (0.60–2.87)
AA	1.14 (0.24–5.46)	0.48 (0.09–2.71)	<0.01 (<0.01–NA)	<0.01 (<0.01–NA)	1.08 (0.22–5.25)	0.89 (0.14–5.49)
Dominant (GG vs. AG+AA)	1.27 (0.68–2.38)	0.91 (0.48–1.72)	1.51 (0.70–3.29)	2.09 (0.77–5.68)	0.94 (0.48–1.83)	1.26 (0.59–2.67)
Recessive (GG+AG vs. AA)	1.03 (0.22–4.81)	0.49 (0.09–2.69)	<0.01 (<0.01–NA)	<0.01 (<0.01–NA)	1.11 (0.23–5.29)	0.80 (0.13–4.84)
G allele	1 (Reference)					
A allele	1.18 (0.70–1.97)	0.87 (0.51–1.47)	1.11 (0.59–2.09)	1.40 (0.65–3.03)	0.97 (0.56–1.68)	1.14 (0.61–2.13)

rs9803799						
TT	1 (Reference)					
GT	1.17 (0.42–3.24)	0.83 (0.29–2.43)	0.84 (0.22–3.31)	1.82 (0.42–7.92)	0.45 (0.14–1.51)	0.50 (0.15–1.62)
GG	0.99 (0.06–16.53)	0.95 (0.05–16.76)	<0.01 (<0.01–NA)	<0.01 (<0.01–NA)	3.06 (0.18–52.68)	1.38 (0.07–26.18)
Dominant (TT vs. GT+GG)	1.15 (0.43–3.03)	0.85 (0.31–2.33)	0.71 (0.19–2.68)	1.64 (0.39–6.99)	0.57 (0.19–1.72)	0.57 (0.19–1.72)
Recessive (TT+GT vs. GG)	0.98 (0.06–16.27)	0.97 (0.06–17.00)	<0.01 (<0.01–NA)	<0.01 (<0.01–NA)	3.24 (0.19–55.53)	1.46 (0.08–27.28)
T allele	1 (Reference)					
G allele	1.12 (0.46–2.74)	0.87 (0.34–2.19)	0.62 (0.18–2.20)	1.45 (0.37–5.68)	0.71 (0.26–1.92)	0.65 (0.24–1.78)

rs2746342						
GG	1 (Reference)					
GT	1.49 (0.75–2.99)	1.63 (0.79–3.34)	0.99 (0.41–2.36)	2.87 (0.85–9.72)	1.39 (0.66–2.95)	1.94 (0.84–4.49)
TT	1.23 (0.47–3.22)	1.35 (0.50–3.66)	1.87 (0.59–5.93)	1.05 (0.16–6.77)	1.60 (0.58–4.43)	1.33 (0.43–4.11)
Dominant (GG vs. GT+TT)	1.43 (0.73–2.78)	1.56 (0.79–3.11)	1.15 (0.50–2.63)	2.39 (0.72–7.87)	1.44 (0.70–2.95)	1.77 (0.80–3.92)
Recessive (GG+GT vs. TT)	0.96 (0.40–2.27)	0.99 (0.41–2.42)	1.89 (0.68–5.25)	0.52 (0.10–2.63)	1.30 (0.53–3.20)	0.89 (0.33–2.44)
G allele	1 (Reference)					
T allele	1.16 (0.74–1.81)	1.22 (0.77–1.93)	1.27 (0.73–2.21)	1.23 (0.61–2.48)	1.26 (0.79–2.02)	1.25 (0.74–2.13)

*P* < 0.05 is considered significant.

CI, confidence interval; CrSr, serum creatinine; eGFR, estimated glomerular filtration rate; FPG, fasting plasma glucose; HbA1c, hemoglobin A1c; NA, not available; OR, odds ratio; WC, waist circumference.

## Discussion

Several studies have revealed the physiological functions of AMPK. AMPK comprises 3 groups, which are AMPKα, β, and γ, and has been studied as a target for T2DM therapy [19]. The role of AMPK in reducing T2DM risk has been discovered. AMPK has a role in glucose uptake in skeletal muscle, suppressing lipogenesis, protein synthesis, lipolysis, stimulating anti-inflammatory effects, and inhibition of gluconeo-genesis [15, 34, 35]. Therefore, a mutation in AMPK might induce susceptibility of T2DM. *PRKKA2* encodes AMPKα2 as one of the active forms of AMPK related to hyperglycemia and insulin resistance [18, 36].

To our knowledge, this is the first study to investigate the association of *PRKAA2* genetic variations (rs2796498, rs9803799, and rs2746342) with clinical characteristics of patients newly diagnosed with T2DM in an Indonesian population. Notably, our results indicated that those participants actually need dual therapy as initial therapy because the mean HbA1c was >9% (ADA). It is common to detect hypertensive problems in patients newly diagnosed with T2DM. However, hypertensive participants among patients newly diagnosed with T2DM tend to have lower risk of albuminemia and left ventricular hypertrophy than T2DM detection among patients newly diagnosed with hypertension [37]. Previous studies found obesity is common among patients newly diagnosed with T2DM [38]. While our participants were mostly women, BMI is a better tool to indicate the association between obesity and T2DM [39].

Genotype frequencies of rs2796498, rs9803799, and rs2746342 in our findings were in Hardy–Weinberg equilibrium. Therefore, those genetic variations remain relatively constant in our participant population [40], although there were low frequencies in wild-type rs2796498 and rs980799.

Of note, our study failed to discover any significant associations of FPG, HbA1c, serum creatinine, eGFR, blood pressure, or obesity status with genetic variations in rs2796498, rs9803799, or rs2746342. Several studies have investigated an association between *PRKAA2* genetic variations and susceptibility to T2DM, but limited studies have observed their association with clinical characteristics. Shen et al. observed an association between *PRKAA2* genetic variations (rs2746342 and rs2143754) and susceptibility to T2DM. Only rs2746342 was reported to have an association with T2DM risk in a Han Chinese population [24].

Similarly, Li et al. proposed that rs2746342 is associated with T2DM risk in a haplotype model, especially with increasing nephropathy. In addition, they studied rs2796498 and suggested it was significantly associated with susceptibility to T2DM [25]. Previously, rs9803799 was found to be correlated with metformin effectiveness [27]. However, only Shen et al. reported the *PRKAA2* genetic variations were associated with clinical characteristics. There was a mean difference of FPG between the dominant and recessive models [24].

Even though AMPKα2 is correlated with hyperglycemia, our study could not ascertain the association of this genetic variation with FPG and HbA1c as glycemic indicators. Therefore, our findings suggest that we still could not combine glycemic indicators and genetic variation analysis as a diagnostic tool for T2DM in our population. Most notably, we found that our study's major allele is a risk factor of T2DM as shown in our previous study. Therefore, our results confirmed previous findings related to the association of *PRKAA2* genetic variations with the susceptibility of T2DM because all of our participants were patients with T2DM.

We could not detect any association of these genetic variations with declining renal function (eGFR <60 mL/min/1.73 m^2^) nor elevated blood pressure as a common comorbidity in patients with T2DM. The absence of apparent association might be caused by our study's recruitment of patients newly diagnosed T2DM. Progressive declining renal function and elevated blood pressure among patients with T2DM depend on T2DM duration [41, 42]. It is possible that for patients newly diagnosed with T2DM, as in our patient population, renal function has not yet changed, and blood pressure remains controlled. AMPK has a unique role in diabetic nephropathy by influencing metabolic memory, podocytes, proximal tubule cells, and fibrosis [43]. The findings that rs2746342 had the highest OR for renal function after adjusting for sex and age warrants further investigation. AMPK has been well-studied in causing arterial dilatation by SERCA and BK_CA_ channels in vascular smooth muscle [44].

Our study did not find any significant association of *PRKAA2* genetic variations and obesity status based on BMI. Because it is well-known that AMPK has a significant role in lipid regulation [34], it should be associated with obesity. Similarly, results were found in studies of Japanese and populations with European and Scandinavian ancestry that showed there was an absence of association of *PRKAA2* genetic variations and BMI [21, 36].

We suggest that the lack of association between *PRKAA2* variations and clinical characteristics in patients with T2DM as shown in our present study is because of their various ancestries. To our knowledge, this study is the first of its kind examining Indonesians, whereas the other studies observed Han Chinese and other populations. In addition, clinical characteristics are not solely the result of gene variation. They could also be influenced by gene–environment interaction [45]. Diet, physical activities, and access to health care facilities might be additional important factors affecting this apparent discrepancy [12, 46]. It could not be denied that access to health care facilities affects uncontrolled blood glucose level and development of T2DM complications. Indonesia is an archipelago, where the access to health care services varies from one area to another [47, 48]. In our study, we included patient participants from 10 different PHC, which have different patient accessibility.

The present study is limited, first, by our relatively small sample size, and the findings should be confirmed using a larger sample. Second, we did not examine other factors that could influence clinical characteristics, such as diet, physical activities, and medication adherence. Third, we recognize that there is genetic heterogeneity in the Indonesian population. Accordingly, to reduce this heterogeneity, we conducted the study only in Yogyakarta where the majority of the people are Javanese. Therefore, in light of our study's limitations, readers should be cautious when generalizing our findings.

## Conclusions

*PRKAA2* genetic variations (rs2796498, rs9803799, and rs2746) are unlikely to be associated with clinical characteristics of patients newly diagnosed with T2DM in our mainly Javanese patient population. Further studies with a larger sample of Indonesians with other specific ethnicities are required to discover the association of these SNPs with clinical characteristics.
